# Evolution of genomic sequence inhomogeneity at mid-range scales

**DOI:** 10.1186/1471-2164-10-513

**Published:** 2009-11-05

**Authors:** Ashwin Prakash, Samuel S Shepard, Jie He, Benjamin Hart, Miao Chen, Surya P Amarachintha, Olga Mileyeva-Biebesheimer, Jason Bechtel, Alexei Fedorov

**Affiliations:** 1 Program in Cardiovascular & Metabolic Diseases Track, Biomedical Sciences, University of Toledo Health Science Campus, Toledo, OH 43614, USA; 2 University of Toledo, Department of Biology, Toledo, Ohio, USA; 3 Dept of Medical Microbiology & Immunology, Infection, Immunity & Transplantation Track, University of Toledo, Health Science Campus, Toledo, OH 43614, USA; 4 Department of Biological Sciences, Bowling Green State University, Bowling Green, OH - 43403, USA; 5 Department of Civil Engineering, University of Toledo, Toledo, Ohio, USA; 6 Program in Bioinformatics and Proteomics/Genomics, University of Toledo Health Science Campus, Toledo, OH 43614, USA; 7 Department of Medicine, University of Toledo, Health Science Campus, Toledo, Ohio, USA

## Abstract

**Background:**

Mid-range inhomogeneity or MRI is the significant enrichment of particular nucleotides in genomic sequences extending from 30 up to several thousands of nucleotides. The best-known manifestation of MRI is CpG islands representing CG-rich regions. Recently it was demonstrated that MRI could be observed not only for G+C content but also for all other nucleotide pairings (e.g. A+G and G+T) as well as for individual bases. Various types of MRI regions are 4-20 times enriched in mammalian genomes compared to their occurrences in random models.

**Results:**

This paper explores how different types of mutations change MRI regions. Human, chimpanzee and *Macaca mulatta *genomes were aligned to study the projected effects of substitutions and indels on human sequence evolution within both MRI regions and control regions of average nucleotide composition. Over 18.8 million fixed point substitutions, 3.9 million SNPs, and indels spanning 6.9 Mb were procured and evaluated in human. They include 1.8 Mb substitutions and 1.9 Mb indels within MRI regions. Ancestral and mutant (derived) alleles for substitutions have been determined. Substitutions were grouped according to their fixation within human populations: fixed substitutions (from the human-chimp-macaca alignment), major SNPs (> 80% mutant allele frequency within humans), medium SNPs (20% - 80% mutant allele frequency), minor SNPs (3% - 20%), and rare SNPs (<3%). Data on short (< 3 bp) and medium-length (3 - 50 bp) insertions and deletions within MRI regions and appropriate control regions were analyzed for the effect of indels on the expansion or diminution of such regions as well as on changing nucleotide composition.

**Conclusion:**

MRI regions have comparable levels of de novo mutations to the control genomic sequences with average base composition. De novo substitutions rapidly erode MRI regions, bringing their nucleotide composition toward genome-average levels. However, those substitutions that favor the maintenance of MRI properties have a higher chance to spread through the entire population. Indels have a clear tendency to maintain MRI features yet they have a smaller impact than substitutions. All in all, the observed fixation bias for mutations helps to preserve MRI regions during evolution.

## Background

The protein coding sequences of humans and of most other mammals represent less than 2% of their genomes. The remaining 98% is made up of 5'- and 3'-untranslated regions of mRNAs (<2%), introns (~37%), and intergenic regions (~60%) [1]. These vast non-protein coding genomic areas, previously frequently referred to as "junk" DNA, contain numerous functional signals of various origin and purpose. They include thousands of non-protein coding RNAs [2], numerous gene expression regulatory signals that surround each gene, chromatin folding structures which include nucleosome positioning sites and scaffold/matrix attached regions [3,4]. These functional DNA regions are non-random in their genomic sequence. The non-randomness or inhomogeneity of base composition has been described at different levels of complexity and sequence length. Starting on the short scale, inhomogeneity occurs in the non-random associations of neighboring bases with each other [5], through the over and under-abundance of particular "words" (usually 5-10 base long oligonucleotides) [6] or longer stretches of DNA, also known as "pyknons" (~18 bases long) [7,8], and up to large regions that cover hundreds of thousands of nucleotides [9]. Compositional inhomogeneity is known to exist in all kinds of species from bacteria to human. However, the particular arrangement of such sequence patterns is often species-specific [10].

It has been the focus of our research to elucidate the genomic sequence non-randomness that we call Mid-Range Inhomogeneity or MRI [11]. We define MRI to be genomic regions from 30 bp to several thousand nucleotides with particular nucleotide enrichments. For large mammalian genomes, there is a high probability that a random sequence of length 20 nucleotides will be unique. Thus, for examining mid-range genomic signals we do not look at particular "words" but only the overall compositional content of particular base(s) that we refer to as *X *(*X *could be a single nucleotide A, G, C, or T or any of their combinations like A+C, or G+T+C). We created a public Internet resource, "Genomic MRI" to study the distribution of *X*-rich regions in any sequence of interest. It was demonstrated that *X*-rich MRI regions are highly overrepresented in mammalian genomes for all kinds *X*-contexts. Particular properties of MRI have also been investigated previously by Mrazek and Kypr [12] and also by Nikolaou and Almirantis [13]. This paper studies the effect of mutations on the evolution of MRI regions in primates.

## Results

### Substitution and polymorphism inside MRI regions

**Figure 1:**
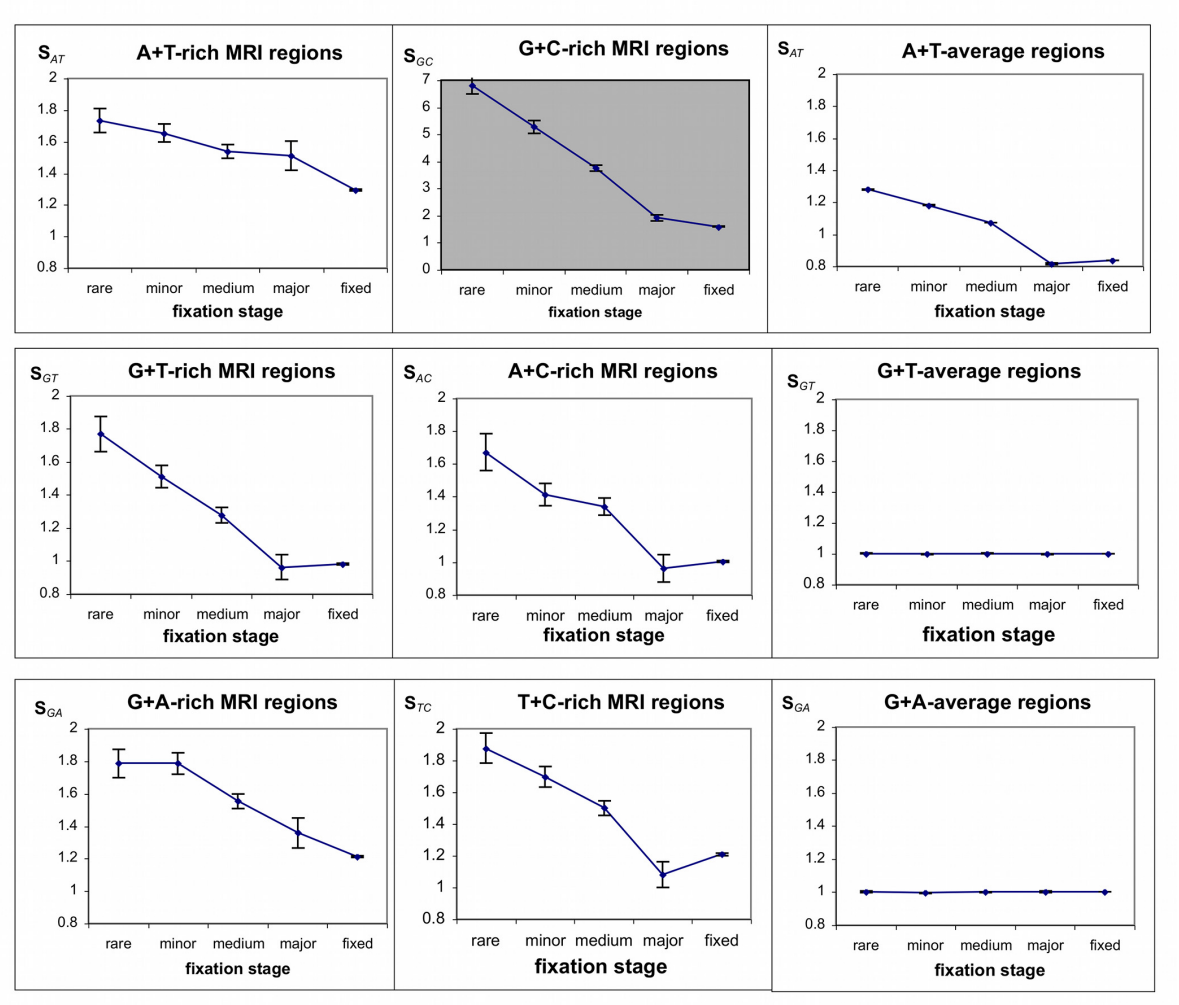
**Substitution Rates in MRI Regions for a Combination of Nucleotides**. For each *X *MRI region--where *X *is for GC-, GT-, or GA-rich or poor regions--the *X*-base composition rate of change is given for all substitutions at different levels of fixation within the human population. The rate of change (*S*_*X*_) is the ratio of *X *to *nonX *substitutions over *nonX *to *X *substitutions in those particular *X*-rich regions. Thus, a ratio of 1 means no change in the *X*-richness of the region whereas a ratio greater than 1 implies degradation of the *X*-rich region and less than 1 implies enrichment of the *X*-rich MRI region. Note that in the control *X*-average regions the *S*_*X*_-ratio is always inverse to *S*_*nonX*_-ratio (*S*_*X *_= 1/*S*_*nonX*_). Therefore, only one graph for each *S*_*X *_and *S*_*nonX *_pair is presented. Since there are significant variations in *S*_*X*_-ratios for different *X *compositions, the graphs are presented in two different scales. The white background presents changes of *S*_*X*_-ratios in the 0.8 to 2 range, while the gray background presents changes in the 0 to 7 range. Vertical bars show the standard error of the means (see Methods section).

**Figure 2:**
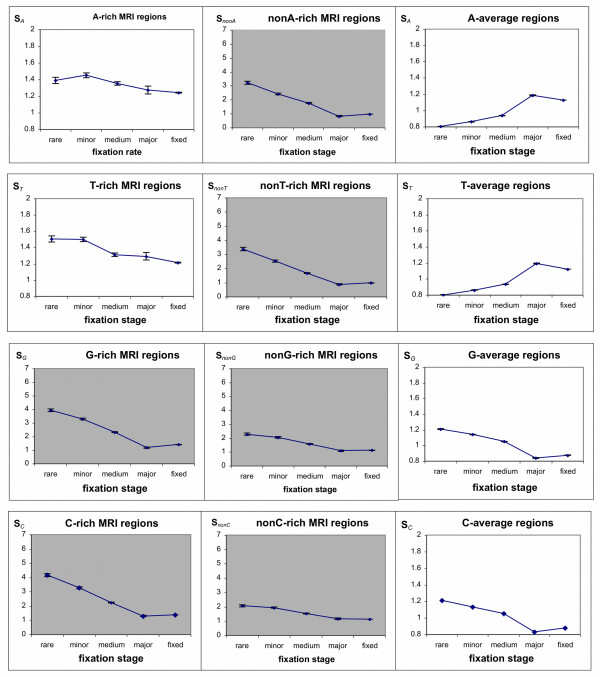
**Substitution Rates in MRI Regions for Single Nucleotides**. For each *X *MRI region--where *X *is for A-, T-, G-, or C-rich or poor regions--the *X*-base composition rate of change is given for all substitutions at different levels of fixation within the human population. The rate of change (*S*_*X*_) is the ratio of *X *to *nonX *substitutions over *nonX *to *X *substitutions in those particular *X*-rich regions. Thus, a ratio of 1 means no change in the *X*-richness of the region whereas a ratio greater than 1 implies degradation of the *X*-rich region and less than 1 implies enrichment of the *X*-rich MRI region. Note that in the control *X*-average regions the *S*_*X*_-ratio is always inverse to *S*_*nonX*_-ratio (*SX *= 1/*S*_*nonX*_). Therefore, only one graph for each *SX *and *S*_*nonX *_pair is presented. Since there are significant variations in *S*_*X*_-ratios for different *X *compositions, the graphs are presented in two different scales. The white background presents changes of *S*_*X*_-ratios in the 0.8 to 2 range, while the gray background presents changes in the 0 to 7 range. Vertical bars show the standard error of the means (see Methods section)

¿From the whole-genome human-chimp-macaque alignment we extracted all the aligned sequences with inhomogeneous nucleotide compositions that satisfy the criteria for MRI (so-called *X*-rich MRI regions; see the Materials and Methods section) and also control regions with nucleotide compositions equal to the average values for the entire human genome. We used the default MRI region length of 100 nucleotides for all computations. Only SNPs located within these MRI and control regions were studied. We particularly focused on the single nucleotide substitutions that maintain or erode MRI features. For example, in GT-rich MRI regions we counted the total number of novel polymorphisms that erode the feature, i.e. G or T → C or A substitutions, denoted as *N*_*GT→CA *_and also the total number of those that maintain the MRI features, i.e. C or A → G or T substitutions, denoted as *N*_*CA→GT*_. In addition, the entire set of recent human substitutions; that is, those nucleotides that differed in human but were the same in chimp and macaque, were processed for the MRI and control regions and presented as "fixed substitutions". The substitution ratio, *S*_*X *_(recall that: *S*_*X *_= *N*_*X→nonX*_/*N*_*nonX→X*_) for the numbers of substitutions that maintain and erode *X*-rich MRI features was calculated for each substitution subtype (rare, minor, medium, and major SNPs and 'fixed'--refer to the Methods section for a detailed explanation) and presented in Figures 1 and 2. With respect to *X*-rich or poor MRI regions, the *X *in Figure 1 represents a two base combination such as GC, AG, GT, etc. while in Figure 2*X *can be any single nucleotide, e.g., A, T, C, and G. If the *S*_*X*_-ratio is equal to 1 the *X*-rich region does not tend towards a change in its *X*-base composition. When *S*_*X *_> 1, the substitutions reduce the *X*-richness of the examined regions, whereas when *S*_*X *_< 1, substitution rates elevate the *X*-richness of the regions. Figures 1 and 2 demonstrate clear linear trends for *S*_*X*_-ratios with respect to increasing fixation of substitutions within human populations.

For the cases of GT-, AC-, AG-, and TC-rich MRI regions (Figure 1), all *S*-ratios for rare SNPs are close to 1.8 (showing erosion of the MRI features). For major SNPs and fixed mutations the *S*_*GT *_and *S*_*AC*_-ratios reach 1.0 (which means no change in the corresponding base composition) and *S*_*GA *_and *S*_*CT*_-ratios reach 1.2 respectively. As for the corresponding control GT-, AC-, AG-, and TC-average regions (all having 50% of corresponding base composition) these lines are flat with all *S*-ratios equal to 1. The latter result is highly expected because of the symmetry of (+) and (-) chromosomal strands for these particular base compositions. Figure 1 also demonstrates that in GC-rich MRI regions the *S*_*GC*_-ratio change has the highest slope from 7.0 for rare SNPs to 1.6 for fixed substitutions. In AT-rich MRI regions (also referred to as nonGC-rich in the tables) the change of *S*_*AT*_-ratio has the lowest slope starting from 1.7 (rare SNPs) and ending at 1.3 (fixed substitutions). The control regions with the average GC/AT compositions (40-42% GC and 58-60% AT) also demonstrate a clear change of S-ratios during substitution fixation. In the control GC-average regions, rare SNPs favor increasing AT-richness (*S*_*GC*_-ratio of 1.3) whereas fixed mutations demonstrate the opposite effect (*S*_*GC*_-ratio of 0.8).

The data for the *S*-ratios for single nucleotides (Figure 2) are very similar to the trends seen in GC- and AT-rich regions. As expected from (+/-) strand symmetry, *S*_*G*_-ratios are equal to *S*_*C*_-ratios and represent about a half of the GC trend. The minor differences between G- and C-rich regions are within the errors of measurement. In the same way the *S*_*A*_-ratios are seen to be the same as the *S*_*T*_-ratios and they comprise approximately half of the effect seen for AT-rich regions.

Based on the observed *S*_*X*_-ratios and the current percentage of *X *bases in the genomic regions under investigation, we calculated the projected equilibrium composition representing the future *X*-composition toward which the examined substitution rates drive these regions. In other words, the equilibrium *X*-composition shows the future level of *X*-richness that would be approached if the *S*_*X*_-ratio as it is observed now were maintained indefinitely. The computed equilibria for each subgroup of substitutions are presented in Table 1. For instance, in the GA-rich regions (G+A composition of 70%), rare SNPs drive GA-richness of these MRI regions down to an equilibrium of 56.6%, while nearly fixed or fixed substitutions drive the GA-composition only to the 65.8% level. For each type of *X*-rich MRI, there is a trend toward minimizing the damage of mutations and preserving the MRI feature as the fixation of the observed substitutions increases. The highest preservation effect is seen for GT- and AC-rich regions (with an observed *X*-base composition of 70%), where the equilibria for fixed substitutions reach about the same level of 70%. For the rest of the types of MRI regions, their equilibria composition is a little below the currently observed base composition.

**Table 1:** Projected *X*-Equilibria.

Equilibrium for *X*-percentage computed from each substitution rate						
**Type of region**	**Observed *X*-percentage**	**rare SNPs**	**minor SNPs**	**Medium SNPs**	**major SNPs**	**fixed substit**.

G-rich	40%	14.5%	16.9%	22.3%	36.0%	32.1%
nonG-rich	7%	14.6	13.4	10.6	7.6	7.8
G-average	20%	17.1	18.0	19.2	23.0	22.2

C-rich	40%	13.8	16.9	23.0	33.90	32.4
nonC-rich	7%	14.5	12.7	10.2	8.0	7.8
C-average	20%	17.1	18.1	19.2	23.1	22.1

A-rich	49.5%	41.4	40.3	42.0	43.5	44.1
nonA-rich	12.9%	32.3	26.3	20.8	10.9	12.6
A-average	29.4%	34.1	32.6	30.7	26.0	27.0

T-rich	49.5%	39.5	39.5	42.8	43.2	44.6
nonT-rich	12.9%	33.4	27.3	19.9	11.5	12.6
T-average	29.4%	34.2	32.6	30.8	25.9	27.1

GT-rich	69.8%	56.9	60.7	64.6	70.8	70.4
nonGT-rich	30.1%	41.7	37.7	36.5	29.2	30.1
GT-average	50.0%	49.9	50.0	50.0	50.0	50.0

GA-rich	70.0%	56.6	56.6	60.0	63.2	65.8
nonGA-rich	29.9%	44.6	42.1	39.1	31.7	34.1
GA-average	50.0%	49.9	50.1	50.0	49.9	49.9

GC-rich	71.3%	26.4	31.7	39.5	56.1	60.6
nonGC-rich	20.0%	30.2	29.3	27.8	27.4	24.4
GC-average	40.7%	34.9	36.8	39.0	45.7	45.0

The calculated equilibria percentages (see Equation 3) for *X*-bases in *X*-rich MRI and control regions with average *X*-composition. Projected equilibria are given based on the substitution rates of rare, minor, medium, and major SNPs as well as for the fixed substitution rates (chimp-macaque to human).

In order to estimate mutation rates for MRI regions versus their respective control regions, we counted the occurrence rates for rare SNPs. The frequency ratio of rare SNPs in MRI rich regions to those in the control regions was calculated. The smallest ratio observed was for A+C content (0.464). This means the frequency of rare SNPs within MRI AC-rich regions is approximately half that of control regions. The highest occurrence ratio for rare SNPs was observed in G- and C-rich MRI regions (1.16 and 1.17 respecitvely). Thus, the occurrence rates of rare SNPs is slightly lower in MRI regions than in the corresponding control regions with the exception of G- and C-rich MRI regions. The entire dataset for the SNPs occurrences in MRI and control regions is presented in Additional file 1. The prevalence of rare and minor SNPs over major SNPs was also observed, their proportion over every MRI and control regions being 5.79.

### Insertions and deletions inside MRI regions

Using the same computational approach as for substitutions, we analyzed human-chimp-macaque triple alignments for the characterization of *indels *(insertions & deletions) that occurred in the human genome during the last 10 million years after the divergence of *H. sapiens *and *P. troglodytes *species. We particularly investigated how indels change the nucleotide composition of MRI regions and control regions with average nucleotide composition. The complete set of data representing short indels (whose sizes are less than three nucleotides) and medium indels (whose sizes are from three to fifty nucleotides) is presented in Additional file 2 (S2A--contains MRI for combinations of nts; S2B--MRI for individual nts). Large indels with sizes over 50 bp were not examined since they are comparable with the sizes of MRI regions and, thus, compromise proper characterization of MRI. The summary data on the influence of both short and medium indels on the composition of MRI and control regions are presented in the Tables 2 and 3 (Table 2 shows MRI regions where *X *represents any single nucleotide; Table 3 is for when *X *represents any combination of 2 nucleotides). For each type of *X*-rich and *X*-control regions the total number of inserted and deleted *X *and *nonX *nucleotides have been computed: *N*_*ins*(*X*)_, *N*_*ins*(*nonX*)_, *N*_*del*(*X*)_, *N*_*del*(*nonX*)_. Finally, the net change in *X *and *nonX *compositions due to indels have been calculated using the following formulas:

**Table 2:** Impact of Indels on *X*-rich MRI Regions, with *X *Representing Any Single Base. The impact of indels on *X*-rich MRI regions and on *X*-average regions, where *X *is for A-, T-, C-, or G-rich or poor. For each particular region we give the total length of examined regions in mega-bases, the percentage composition or content of *X*, the number of changes in *X *due to insertions and deletions (Δ*X *= *N*_*ins*_(*X*) - *N*_*del*_(*X*)), and the change in *X *composition due to both indels and substitutions.

	A-rich	nonA-rich	A-average
total length	66.9 Mb	72.4 Mb	800.4 Mb
content of A	49.6%	12.9%	30.5%
ΔA	-16850	-7390	-44182
ΔnonA	-24748	-29257	-98769
net A% change INDEL	0.006%	-0.004%	-0.0001%
net A% change SUBST	-0.027%	-0.002%	-0.014%

	T-rich	nonT-rich	T-average

total length	67.8 Mb	71.1 Mb	800.4 Mb
content of T	49.5%	13.1%	30.5%
ΔT	-21849	-7078	-47238
ΔnonT	-24084	-22716	-97057
net T% change INDEL	0.001%	-0.004%	-0.0004%
net T% change SUBST	-0.024%	-0.002%	-0.013%

	G-rich	nonG-rich	G-average

total length	52.0 Mb	60.4 Mb	884.7 Mb
content of G	40.10%	7.20%	20.40%
ΔG	-1185	-7080	-31780
ΔnonG	-12864	-37512	-139126
net G% change INDEL	0.009%	-0.006%	0.0003%
net G% change SUBST	-0.052%	0.009%	0.016%

	C-rich	nonC-rich	C-average

total length	52.0 Mb	60.4 Mb	883.9 Mb
content of C	40.10%	7.20%	20.50%
ΔC	-829	-6700	-33823
ΔnonC	-12418	-35277	-140331
net C% change INDEL	0.009%	-0.006%	0.0002%
net C% change SUBST	-0.049%	0.009%	0.015%

**Table 3:** Impact of Indels on MRI Regions, with *X *Representing Combinations of Any Two Bases.

	GC-rich	nonGC-rich	GC-average
total length	17.8 Mb	54.8 Mb	780.6 Mb
content of GC	71.00%	20.30%	40.90%
ΔGC	1405	-9100	-31622
ΔnonGC	-765	-5951	-56278
net GC% change INDEL	0.005%	-0.011%	0.001%
net GC% change SUBST	-0.094%	0.042%	0.034%

	GT-rich	nonGT-rich	GT-average

total length	34.9 Mb	34.6 Mb	1192 Mb
content of GT	69.10%	30.90%	50.00%
ΔGT	-8278	-6837	-121644
ΔnonGT	-4518	-8502	-120128
net GT% change INDEL	0.002%	-0.006%	-0.0001%
net GT% change SUBST	0.004%	0.001%	-0.0003%

	GA-rich	nonGA-rich	GA-average

total length	69.2 Mb	70.0 Mb	978.3 Mb
content of GA	69.75%	30.22%	49.99%
ΔGA	-23641	-13935	-96617
ΔnonGA	-14185	-28480	-100013
net GA% change INDEL	0.004%	-0.002%	0.0002%
net GA% change SUBST	-0.014%	0.014%	0.0002%

The impact of indels on *X*-rich MRI regions and on *X*-average regions, where *X *is for GC-, GT-, or GA-rich or poor. For each particular region we give the total length of examined regions in mega-bases, the percentage composition or content of *X*, the number of changes in *X *due to insertions and deletions (Δ*X *= *N*_*ins*_(*X*) - *N*_*del*_(*X*)), and the net change in *X *composition due to both indels and substitutions.

Tables 2 and 3 demonstrate that in the human genome there is a prevalence of deletions over insertions (i.e. negative values of Δ*X *and Δ*nonX*) for every type of nucleotide content studied and for every type of MRI and control region with the exception of GC-indels in GC-rich MRI regions. In the last case ΔGC is positive and equal to 1405 added nucleotides (over a total set of 1.8 million nucleotides). For all other cases of *X *except *X *= GC, short and medium indels cause gradual contraction of genomic regions in humans. This means that there is no nucleotide composition equilibrium to which the indels drive the genome in the indefinite future and, therefore, these equilibria have not been calculated. Table 2 shows that, for every *X*-rich region, indels result in the increasing the richness of corresponding MRI regions (positive net *X*% change for *X*-rich region and negative net *X*% change for *nonX*-rich region). In all *X*-control regions the net *X*% change is several times less than in the corresponding *X*-rich and *nonX*-rich regions.

Finally, we calculated the percentage of nucleotide composition changes in case of both substitutions and indels separately, that occurred in the human genome during last ten million years after the divergence of human and chimpanzee. These results are presented in Tables 2 and 3 and serve to measure the relative importance of substitutions versus indels to the nucleotide composition of MRI regions.

## Discussion

Consistent with Chargaff's second parity rule [14], both the G or C base content of the human genome are equal to 21.1%, while A or T comprise 28.9% each. However, in thousands and thousands of genomic regions of various lengths, the composition of A, T, C, or G content (or different combinations of these bases) exist at extremes quite different from the aforementioned averages. De novo mutations constantly occur in populations and could dramatically change the base composition of a genomic region during the course of evolution. A good choice for a large-scale computational analysis of these novel mutations is in the examination of 'rare' single-nucleotide polymorphisms (SNPs, or mutations that are present only in a small group of individuals and absent in a majority of the population). Rare SNPs are mutations that have recently occurred. However, even among rare SNPs there exists a minor subgroup of "older" mutations that have diminished their frequency to rare events. The relative size of this subgroup is in reverse proportion to the effective size of the population [15], and hence, it represents only a minor fraction of the recent mutations for humans. Here we show that rare SNPs in genomic regions with average nucleotide composition are enriched by G or C → T or A substitutions that drive the genomic composition of those regions to a level of 35% for G+C and 65% for A+T. On the other hand, examining the same regions for mutations that have substantially propagated into human populations (i.e. medium and high frequency SNPs as well as "fixed" recent mutations) demonstrates that these fixed or nearly fixed substitutions are much less prone to G or C → T or A changes. Instead, high frequency SNPs as well as fixed substitutions tend to drive genomic regions with average base composition to 45% G+C composition.

Here we have focused particularly on the influence of mutations on the evolution of specific genomic regions with strongly inhomogeneous base compositions that are far from the average distribution of nucleotides (so-called MRI regions where G+C, G+A, C+T, G+T, or A+C composition is at least 70%, A+T composition is above 80%, or single base frequency reaches nearly 50%). For all types of MRI regions, we found that novel substitutions (rare SNPs) tend to more strongly erode the compositional extremes (*X*-richness) of the region. At the same time, these mutations undergo a strong fixation bias during their propagation into populations in such a way that fixed substitutions tend to preserve MRI regions. For example, rare SNPs inside GC-rich MRI regions drive the nucleotide composition of those regions to the 26% GC level. However, fixed substitutions in the same GC-rich MRI regions drive GC composition only to 61%. The highest fixation was seen for GT- and AC-rich MRI regions, which preserves the current GT- and AC-composition of 70%.

This trend of preserving nucleotide composition of MRI regions with respect to the increasing fixation of substitutions could be explained by at least two different mechanisms. First, one could observe that there are some important functional roles for MRI regions. For instance, GC-rich MRI regions include well-known CG-islands, prominent regulators for gene expression [16,17]. Thus, these regions should be under the constraint of purifying selection, preserving their important features. Other MRI regions may be under similar selective pressure due to association with functional genomic elements and/or, as yet unknown, sequence signals. Second, fixation bias inside MRI regions might be due to some non-symmetry in cellular molecular machinery involving DNA repair, replication, and/or recombination processes. For example, the Biased Gene Conversion (BGC)-theory engages this particular scenario in order to explain the maintenance of CG-rich regions [18,19]. (It must be observed, however, that this theory operates on much larger genomic scales and refers to isochores that cover from hundreds of thousands to millions of bases.) Thus far it is inconclusive as to which of these two scenarios, or a combination thereof, best fits the observed trends. For the case of GC-rich sequences, we conjecture that both scenarios could be taking place to some extent to preserve MRI.

Interestingly, the highest level of MRI erosion for rare SNPs is observed in GC-rich MRI regions. Novel substitutions in these particular regions try to drive GC-content to the lowest level of 26% (see Table 1). We explain this phenomenon via uneven distribution of CpG dinucleotides, which are most abundant in GC-rich MRI regions. It is well known that CpG dinucleotides are extreme hot spots for the C → T and G → A mutations, which cause CpG to be the most underrepresented dinucleotide in vertebrate genomes. Therefore, CG-rich MRI regions, which are known to have the highest concentration of CpG dinucleotides, should have the highest rate of de novo mutations in the direction C or G → T or A. Human SNPs having C/T alleles in the CpG/TpG context with the orthologous chimp allele in the TpG context have an increased error rate of 9.8% for ancestral misidentification (see the Methods section) due to the probability of a coinciding chimp SNP at the same locus [20]. However, since the strength of the mutational erosion in the GC-rich MRI regions is so high, even an error rate of 9.8% will not change the observed trend.

So far we have discussed only the effect of substitutions on the nucleotide composition of mid-range genomic regions. Insertions and deletions are the other types of mutations that change genomic sequences and, therefore, should also be considered. In mammals, short and medium indels are several times less frequent than substitutions. Currently, there is not enough data on human indel SNPs to perform the same analysis of their fixation process as we did for substitutions. For this reason we studied only fixed indels in humans (indels present in human but differing in chimp and macaque). Our examination demonstrated that indels weakly influence the nucleotide content of MRI regions toward preserving their inhomogeneous composition, in the same manner as the fixation bias of fixed substitutions (see Tables 2 and 3).

## Conclusion

The fixation bias on both fixed substitutions and indels tend to protect MRI regions from degradation of their compositional extremes amid the constant flow of random mutations, thus suggesting their contribution in the preservation of functional and structural complexities of the human genome. Future research on these genomic elements as well as refinement of our approach should help determine the extent of maintenance of MRI by natural selection.

## Methods

### Genomic samples and computation of recent human mutations ("fixed substitutions")

Taking human-chimp (human build 36.1 and chimp build 2 version 1) and human-macaque (macaque build v1 edit4) whole-genome pairwise alignments from the UCSC Genome Browser [21]http://hgdownload.cse.ucsc.edu/downloads.html as input, we generated a Perl script for the identification of the common genomic regions for these three species. The process involved the usage of the human genomic sequence as the reference for the location with the chimp and macaque sequences being extracted only in areas where the sequences of all three species were represented. We then invoked the ClustalW (v1.83) program with default parameters to obtain a whole-genome human-chimpanzee-macaque triple alignment. The obtained alignment is available at our website http://bpg.utoledo.edu/human_chimp_macaque.html. This triple alignment was used to calculate the dataset of recent mutations in humans. We considered a recent substitution at a particular position (for example T → C at position 23456719 on chromosome 7) to be valid if the human genome has a C base while both chimp and macaque have a T base in the corresponding aligned positions. In addition, we required that the quality of the alignment in the vicinity of the mutation be reliable (more than 70% similarity between human and macaque in the 20 bp flanking region [-10, +10]). The frequency table of all inferred recent human mutations is presented in the Additional file 3. We analyzed these recent substitutions together with the SNP datasets and call the former mutations "fixed substitutions," assuming that the majority of them occurred less than 10 million years ago and were already fixed across all human populations. In the same manner we processed indels in the triple alignments and computed all unambiguous cases of human insertions and deletions with sizes from 1 to 49 nucleotides.

### Processing of SNP data

Over 4.62 million human SNPs from all chromosomes were obtained (dbSNP build 128 [22], ftp://ftp.ncbi.nih.gov/snp/), filtered for completeness and correctness annotations (676499 records discarded total), and mapped onto the whole-genome human-chimpanzee alignment. SNP allele frequencies were averaged from the frequency data of all populations of that allele. However, only those SNPs that were successfully located within the alignment were processed further. For each SNP site we verified the existence of the particular polymorphic bases in the specified position of the human genome reference sequence and also in the corresponding aligned position on the chimp genomic sequence. If any of these two species had different bases than the SNP alleles, the SNP was discarded (20469 SNPs discarded total).

Otherwise, we defined the origin of the polymorphism based on the chimpanzee nucleotide. Consider the following example to illustrate this process: suppose one has an A/G polymorphism located at position 34567812 of chromosome 5 with an average A allele frequency of 0.6 and a G allele frequency of 0.4. Then at position 34567812 of chromosome 5 of the human genome reference sequence (Genbank build 36.1), we would first examine if the A or G allele is present at that position and discard the SNP if not. Next, using the flanking region of that SNP we could align the chimp genomic sequence. If the chimp nucleotide were T or C then the SNP would also be discarded because those alleles are not a part of the human haplotype at that position. However supposing that the chimp nucleotide were G, then the polymorphism would be declared as a G → A polymorphism with G being declared the ancestral allele that at some point in human evolution mutated into an A allele within some human population(s). From the frequency data we may finally characterize this example SNP more precisely as a 0.4G → 0.6A polymorphism.

Using this approach we successfully characterized 3.93 million human SNPs. This last group of SNPs was divided into four subgroups based on the abundance of the mutant allele in the given human populations:

I. rare polymorphisms with the frequency of the mutated allele being less than 3%;

II. minor polymorphisms with frequencies ranging from 3% to 20%;

III. medium polymorphisms with frequencies going from 20% to 80%; and

*IV. major polymorphisms with the frequency being above 80%*.

For our method, misidentification of the ancestral allele might arise when the site for the human SNP is also polymorphic in chimp populations (e.g. A/G polymorphism) or for the possible case that this site had a recent substitution in chimps (A → G) after their divergence from humans. Human and chimpanzee genomes only differ by 1.23% due to single nucleotide substitutions with 1.06% being due to fixed substitutions and the rest (0.17%) being due to polymorphisms in human and chimp [20]. Moreover, according to the Chimpanzee Sequencing and Analysis Consortium the average estimated error rate of human alleles being misidentified due to chimp polymorphisms is only ~1.6% across all typical SNPs, which is acceptably low. It is also observed, however, that in the mutational hotspot of the CpG dinucleotide, there is an increased error rate for ancestral misidentification. If the human alleles are C/T in the CpG and TpG context and the chimp allele is T (in the TpG context) then the estimated error rate is actually 9.8% [20]. Thus, in the context of studying our MRI regions, any substitution (especially in GC-rich MRI regions since they contain an overabundance of G and C) going from TpG → CpG could have the ancestral allele misidentified, which would mean that the substitution would actually be CpG → TpG, although in the case of GC-rich MRI regions where such dinucleotides are more likely, an error rate of 9.8% is not sufficient to change the trend or conclusion of our results.

### *X*-rich MRI genomic regions and control regions with average base composition

Any base or combination of bases can be described by a parameter *X*. For example, *X *could be G-base; C+T-bases; or A+T+G bases, et cetera. It is also useful to refer to *nonX *base(s) as all bases not *X*. Thus, *X *+ *nonX *must represent all four nucleotides A, G, T, and C. For the examples above, *nonX *are A+T+C-bases; G+A-bases, and C-base, respectively. MRI is characterized by a specific base composition within a region under analysis. We characterize *X*-rich MRI regions based on an overabundance of the *X *base(s) within a region of a certain length (the so-called window), where the percentage of *X *should be above a certain threshold (Bechtel et al 2008). We calculated MRI regions in the human genome for single nucleotides and various nucleotide combinations using a stretchy window of 100+ nucleotides with the following threshold parameters: for A or T the threshold was 49%; for G or C we used 40%; for G+C it was 70%; for A+T the threshold was at 80%; for G+T, C+A, G+A, and C+T were at 70%; nonA or nonT was 87%; and non G or non C the threshold was 93%. These thresholds were chosen experimentally in such a way that MRI regions should represent about 2% of the whole human genome. A stretchy window of *N *+ nucleotides means that we scan genomic sequence with an *N*-size window to find a genomic MRI region that fits the threshold criterion, then we extend the window above the detected region by 10 nt steps until the criterion is no longer met. After registering the full MRI region we jump beyond the current MRI region and continue with the default *N*-size window. Using this approach we characterized all MRI regions in the triple human-chimp-macaque alignments using the human sequence for calculating nucleotide composition and MRI features. We also discarded those MRI regions in the alignments where the indel composition exceeded 50%. For the collection of control regions with average base compositions we used the same stretchy window approach with the nucleotide composition corresponding to the following average genomic frequencies: for A, T between 30 and 31% thresholds; for G, C between 20-21%; for G+C between 40-42%; A+T at 58-60%; G+T, C+A, G+A, or C+T were at 49-51%.

Note that control regions with genome-average *X*-composition also have genome-averaged *nonX*-composition. Therefore, their subsitution ratios are in inverse proportion to each other: *S*_*X *_= 1/*S*_*nonX*_. Due to this only one ratio for *X *and *nonX *pair is shown in Figures 1 and 2.

### Calculation of the substitution ratios in MRI and control regions

Studying SNPs and fixed substitutions in *X*-rich MRI regions we measured the number of changes from *X *to *nonX *(denoted as *N*_*X→nonX*_) and also the number of changes from *nonX *to *X *(denoted as *N*_*nonX→X*_). The fluctuations in the observed distribution of *N*_*X→nonX *_and *N*_*nonX→X *_are well-known as Poisson noise. Thus, the standard deviation for the true values for *N*_*X→nonX *_and *N*_*nonX→X *_is calculated according to *v *the Poisson distribution, that is: *σ*_*N *_= . For each *X*-rich MRI region we measured the substitution *S*_*X*_-ratios with *S*_*X *_= *N*_*X→nonX*_/*N*_*nonX→X *_shown in Figures 1 and 2. The propagation of uncertainty for a ratio *f *= *A/B *can be calculated using the formula (*σ*_*f*_/*f*)^2 ^= (*σ*_*A*_/*A*)^2 ^+ (*σ*_*B*_/*B*)^2 ^- 2(*σ*_*A*_·*σ*_*B*_)/(*A·B*)·*ρ*_*AB*_, where *ρ*_*AB *_is the correlation coefficient for *A *and *B *variables. Because the observed frequency of having a SNP at a genomic site in humans is less than 1%, it is correct to assume that the correlation between *N*_*X→nonX *_and *N*_*nonX→X *_is negligible. Therefore, the standard deviation for the *S*_*X *_ratio was calculated by the following formula:

### Calculation of base composition equilibrium for the observed substitution rates

As described in the previous paragraphs, for studying SNPs and fixed substitutions in *X*-rich MRI regions of the human genome we measured the number of changes from *X *to *nonX *(denoted as *N*_*X→nonX*_) and also the number of changes from *nonX *to *X *(denoted as *N*_*nonX→X*_). These *N*_*X→nonX *_and *N*_*nonX→X *_helped us to estimate the frequencies of these two types of mutations per *X *or *nonX *site, named here as *F*_*X→nonX *_and *F*_*nonX→X*_, correspondingly. Suppose one has a sample of MRI regions with a total nucleotide sequence length of *L *and a composition of *X *with the region richness given as *P*_*X *_being measured in numbers from 0 to 1. Then, the total number of *X *sites in this sample will be *L*·*P*_*X*_, and the total number of *nonX *sites will be *L*·(1 - *P*_*X*_). During a certain time interval called Δ*T *there will be Δ*N*_*X→nonX *_and Δ*N*_*nonX→X *_substitutions. Therefore the frequency of substitutions per site is *F*_*X→nonX *_= Δ*N*_*X→nonX*_/(Δ*T·L·P*_*X*_) and *F*_*nonX→X *_= Δ*N*_*nonX→X*_/(Δ*T·L*·(1 - *P*_*X*_)). It is impossible to measure directly these Δ*N *values for a specific time interval of Δ*T*. However, with a good approximation we can assume that the frequencies are proportional to the observed numbers *N*_*X→nonX *_and *N*_*nonX→X *_and can be represented by the simple formula: *F*_*X→nonX *_= *A·N*_*X→nonX*_/*P*_*X *_and *F*_*nonX→X *_= *A·N*_*nonX→X*_/(1 - *P*_*X*_), where *A *is a scaling factor having the same value for *F*_*X→nonX *_and *F*_*nonX→X*_, since *N*_*X→nonX *_and *N*_*nonX→X *_are counted from the same sample. In a gedanken experiment, let's assume that the current *F*_*X→nonX *_and *F*_*nonX→X *_values will stay unchangeable forever for our MRI sample. Then, in time, mutations should alter the base composition of our sample until it reaches an equilibrium composition with a new percentage for *X*-bases denoted here as *Q*_*X*_. This equilibrium composition *Q*_*X *_can be computed using the observed parameters of *P*_*X*_, *N*_*X→nonX*_, and *N*_*nonX→X*_. Indeed, under the equilibrium, the number of changes from *X *to *nonX *must be equal to the number of reverse changes from *nonX *to *X*, or:(1)

We can compute these Δ*N*_*X→nonX *_and Δ*N*_*nonX→X *_values from frequencies in such a way:

also in a similar way

By putting these transformations into Equation 1 we get:

or(2)

Finally, simple transformation of Equation 2 gives us the final Equation 3 for calculation of equilibrium percentage:

In the Results section, Formula 3 is used to compute the equilibrium percentage for *X*-bases in the studied MRI regions.

## Abbreviations

MRI: mid-range inhomogeneity; SNP: Single nucleotide polymorphisms; Mb: Megabase(s); indels: insertions and deletions; nt: nucleotide(s).

## Authors' contributions

AP, BRH, SPA, MC, JH, OMB were responsible for computational processing of the human-chimp-macaque datasets and creating the described programs. SS was responsible for the procuring and processing of the SNP data from dbSNP. JMB was responsible for the quantification of SNP data in the alignment. AP was also responsible for the processing of fixed substitutions and indel data from the three way alignment. AF supervised the project, provided guidance and wrote the draft. SS and AP also contributed to editing, typesetting, and writing the draft. All authors have read and approved the final manuscript.

## Supplementary Material

Additional file 1 **S1 - SNP dataset**. Complete set of data representing the number of occurrences of major, middle, minor and rare SNPs in all *X *MRI regions (where *X *could represent either a two-base combination [GC, AG, GT, etc.] or a single base) and their corresponding control regions, which have an average nucleotide composition for the respective *X*. Click here for file

Additional file 2 **S2A - Indel data for combination of nts, S2B - Indel data for individual nts**. • 2A - Complete set of data representing the total number of *X *nucleotides (where *X *represents a two base combination such as GC, AG, GT, etc.) inserted or deleted due to short indels, whose sizes are less than three nucleotides in length, or medium indels, whose sizes range from three to fifty nucleotides in length, for all *X *MRI regions, and for the control regions, which have an average nucleotide composition for the respective *X*. • 2B - Complete set of data representing the total number of *X *nucleotides (where *X *represents a single base) inserted or deleted due to short indels, whose sizes are less than three nucleotides in length, or medium indels, whose sizes range from three to fifty nucleotides in length, for all *X *MRI regions, and for the control regions, which have an average nucleotide composition for the respective *X*. Click here for file

Additional file 3 **S3 - Dataset of fixed substitutions**. Complete set of data representing the number of occurrences of fixed substitutions on all *X *MRI regions (where *X *could represent either a two-base combination [GC, AG, GT, etc.] or a single base), and the control regions, which have an average nucleotide composition for the respective *X*. Click here for file
